# Decreased Adiponectin Levels Are a Risk Factor for Cognitive Decline in Spinal Cord Injury

**DOI:** 10.1155/2022/5389162

**Published:** 2022-01-17

**Authors:** Fan-jie Liu, Hong-hao Xu, Ying Yin, Yuan-zhen Chen, Liang-yu Xie, Hua-zhong Li, Dan-dan Wang, Bin Shi

**Affiliations:** ^1^Bone Biomechanics Engineering Laboratory of Shandong Province, Shandong Medicinal Biotechnology Center (School of Biomedical Sciences), Neck-Shoulder and Lumbocrural Pain Hospital of Shandong First Medical University, Shandong First Medical University & Shandong Academy of Medical Sciences, Jinan, 250062 Shandong Province, China; ^2^Department of Trauma Bone Surgery, The Affiliated Hospital of Shandong University of TCM, Jinan, 250014 Shandong Province, China; ^3^Department of Acupuncture, The Affiliated Hospital of Shandong University of TCM, Jinan, 250014 Shandong Province, China

## Abstract

**Objective:**

Spinal cord injury (SCI) has become popular in recent years, and cognitive decline is a common complication. Adiponectin is a common protein hormone involved in the course of many diseases, but its relationship with SCI has not yet been elucidated. The purpose of our prospective study is to explore whether adiponectin can be used as a biomarker of cognitive decline in SCI.

**Methods:**

A total of 64 healthy volunteers and 92 patients with acute SCI were recruited by us. Serum adiponectin levels, demographic data (age and gender), lifestyle (smoking and drinking), medical history (diabetes and hypertension), and clinical baseline data (low-density lipoprotein, high-density lipoprotein, and fasting blood glucose) were recorded. Three months after enrollment, we used the Montreal Cognitive Assessment (MoCA) to evaluate cognitive function. Based on a quarter of the serum adiponectin levels, SCI patients were divided into 4 groups, and the differences in their MoCA scores were compared. In addition, we used multivariate linear regression to predict the risk factors of the MoCA score.

**Results:**

The serum adiponectin level (6.1 ± 1.1 *μ*g/ml) of SCI patients was significantly lower than that of the healthy control group (6.7 ± 0.9 *μ*g/ml), and there was a significant difference between the two (*p* < 0.001). The group with higher serum adiponectin levels after 3 months of spinal cord injury had higher MoCA scores. Multivariate regression analysis showed that serum adiponectin level is a protective factor for cognitive function after SCI (*β* = 0.210, *p* = 0.043).

**Conclusions:**

Serum adiponectin levels can be used as an independent predictor of cognitive function in patients with acute SCI.

## 1. Introduction

Spinal cord injury (SCI) has an epidemic trend worldwide, and the reported incidence is about 3-195 cases/million per year, and the average incidence in different regions is 1/1000 [1]. There are about 20,000 new SCI cases in the USA each year, and there is a lack of relevant data reports in China [2]. The average age of onset of SCI patients is 43 years old, and the most common causes are motor vehicle accidents and falls [3]. The per capita life-long economic burden of SCI patients ranges from US$1.5 million to US$3 million depending on the severity of the disease, and the annual socioeconomic burden of SCI is estimated to be US$2.67 billion [4]. Therefore, there is an urgent need to improve the health and quality of life of patients with SCI.

Adiponectin is a protein hormone derived from adipocytes, which can regulate the metabolism of glucose and fatty acids, and has a wide-ranging regulatory role in multisystem diseases such as endocrine system, musculoskeletal system, nervous system, and atherosclerosis [5]. Adiponectin is a 244-amino acid polypeptide composed of four different domains, and its structure is similar to complement 1Q and TNF-*α* factor [6, 7]. The human adiponectin gene is located on chromosome [8]. In serum, it has the following three forms: trimers, hexamers, and high molecular weight polymers. Similarly, adiponectin also has three main receptors: AdipoR1 (highly expressed in skeletal muscle), AdipoR2, (highly expressed in the liver), and T-cadherin (highly expressed in vascular endothelial cells and smooth muscle) [9]. AdipoR1 and R2 can regulate inflammation and oxidative stress by acting on AMPK and (PPAR)-*α* pathways, while activating T-cadherin can attenuate oxidative stress, thereby protecting vascular endothelial cells from playing an antiatherosclerotic effect [7]. Compared with many other hormones, the concentration of adiponectin in plasma is relatively high, about 5-10 *μ*g/ml [10], which makes it easy to detect and has the advantage of being a potential biomarker.

Cognitive impairment is one of the important complications of SCI, indicating that SCI may increase the risk of cognitive impairment after SCI [11]. Studies have shown that adiponectin and leptin can act synergistically to promote synaptic and memory functions in the brain, while lower levels of adiponectin can cause cognitive decline [12]. Whether adiponectin is related to cognitive impairment after SCI is the question that our study aims to solve. The clarification of the relationship between adiponectin and cognitive impairment is helpful for the diagnosis, treatment, and rehabilitation of SCI patients with cognitive impairment.

## 2. Methods

### 2.1. Study Population

**Figure 1:**
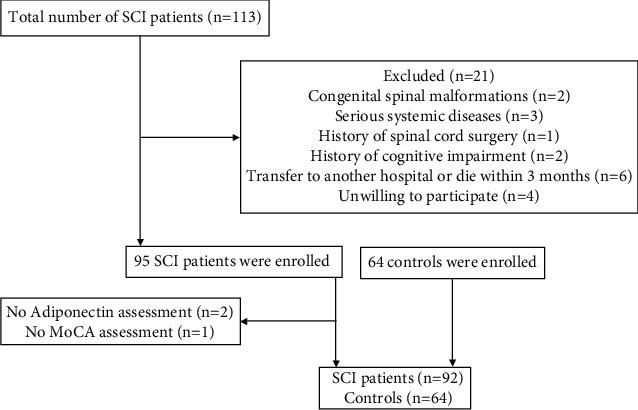
Flowchart of the study. SCI: spinal cord injury; MoCA: Montreal Cognitive Assessment.

The study included all patients with acute SCI who visited Neck-Shoulder and Lumbocrural Pain Hospital and the Affiliated Hospital of Shandong University of TCM between September 2018 and August 2021. The diagnosis of acute SCI is based on diagnosis and treatment guidelines [13, 14]. Exclusion criteria include the combined presence of congenital spinal malformations and serious systemic diseases, history of spinal cord surgery and cognitive impairment, transfer to other hospitals or death within 3 months, and unwilling to receive adiponectin testing or MoCA assessment. In addition, age- and gender-matched populations were recruited as controls. The flowchart is shown in Figure 1. The study strictly complied with the ethical requirements of the Declaration of Helsinki and was approved by the hospital ethics committee. All participants or family members agreed to participate in the study.

### 2.2. Baseline Data Collection

The baseline data we collected includes demographic data (age and gender), lifestyle (smoking and drinking), medical history (diabetes and hypertension), and clinical baseline data (low-density lipoprotein, high-density lipoprotein, and fasting blood glucose). The demographic data, lifestyle, and medical history are obtained by asking patients or family members and medical records. The clinical baseline data comes from routine inspections in the hospital. All data is archived by a dedicated person.

### 2.3. Cognitive Function Test

In this study, the currently popular MoCA scale was used to detect the cognitive function of patients with SCI after 3 months of onset. The MoCA scale began as a cognitive screening tool in 1996. It was created by Ziad Nasreddine in Montreal, so it was named after the place name. The subject was asked to answer 30 questions within 10 minutes, and each question was assigned 1 point, so the total score was 30 points. The average score of a normal person is 27.4, and a score of <26 is considered to have cognitive impairment. MoCA has been translated into 46 languages for free use by scientific researchers and clinicians all over the world [15].

### 2.4. Serum Adiponectin Detection

Blood samples of patients with acute SCI were collected immediately after admission, and the serum samples were stored in a refrigerator at -80°C and were frozen and thawed for use before analysis. Serum adiponectin levels use ELISA (Abcam, USA) reagents, and the operation process is carried out strictly in accordance with the instructions provided by the manufacturer.

### 2.5. Statistical Analysis

The software used in the statistical analysis of this study is SPSS 22.00. *n* is used to record count data, and the mean ± standard deviation is used to represent continuous data. The chi-square test was used for the comparison of the two groups of count data, and the *t*-test was used for the comparison of the two groups of continuous data. The correlation analysis between serum adiponectin level (quartile) and MoCA score was performed by *p* for trend test. Multivariate linear regression analysis was used to finally determine the risk factors that affect the cognitive function of SCI patients. The statistical standards for all the above tests are two-tailed *p* < 0.05.

## 3. Results

### 3.1. Baseline Data

**Figure 2:**
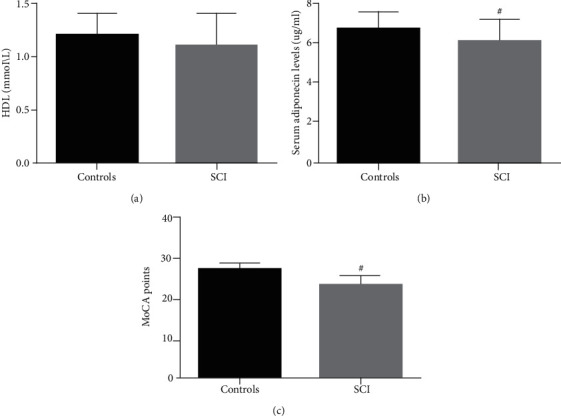
Comparison of HDL, MoCA, and serum adiponectin levels between the two groups. SCI: spinal cord injury; HDL: high-density lipoprotein; MoCA: Montreal Cognitive Assessment.

Based on the principle of voluntary participation, a total of 92 acute CI patients and 64 controls were included in the study after strict screening. The high-density lipoprotein levels of the control group and SCI group were 1.2 ± 0.2 mmol/l and 1.1 ± 0.3 mmol/l, respectively. The control group had a higher level of high-density lipoprotein (*p* < 0.001), and the results are shown in Table 1 and Figure 2(a). Except for high-density lipoprotein, the two groups had no differences in demographic data (age and gender), lifestyle (smoking and drinking), medical history (diabetes and hypertension), and clinical baseline data (low-density lipoprotein and fasting blood glucose) (statistically significant (*p* > 0.05)). The specific data information is shown in Table 1.

### 3.2. Serum Adiponectin Level and MoCA Score

The serum adiponectin levels of the control group and SCI group were 6.7 ± 0.9 *μ*g/ml and 6.1 ± 1.1 *μ*g/ml, respectively. There are significant differences between the groups (*p* < 0.001). The results are shown in Table 1 and Figure 2(b); the MoCA scores of the control group and SCI group are 27.4 ± 1.3 points and 23.3 ± 2.5 points, respectively. The MoCA score of the SCI group was significantly lower than that of the control group (*p* < 0.001). The results are shown in Table 1 and Figure 2(c).

### 3.3. Correlation between MoCA and Serum Adiponectin Levels

According to the serum adiponectin level, we divided patients with acute SCI into 4 groups, namely, *Q*1, *Q*2, *Q*3, and *Q*4 groups. We found that as the serum adiponectin level increased (*Q*1-*Q*4), the MoCA score also increased, and after statistical analysis, this correlation was significant (*p* < 0.001). The above relevant analysis results are shown in Table 2. Therefore, serum adiponectin may be a cognitive protective factor in SCI.

### 3.4. Multivariate Regression Analysis

In order to predict the risk factors affecting the cognitive function of SCI patients, we included serum adiponectin levels, age, gender, smoking, drinking, diabetes, hypertension, low-density lipoprotein, high-density lipoprotein, and fasting blood glucose into the regression model. The analysis results showed that after adjusting age, gender, smoking, drinking, diabetes, hypertension, low-density lipoprotein, high-density lipoprotein, and fasting blood glucose, the serum adiponectin level is still a significant factor affecting the cognitive function of SCI patients (Table 3). Therefore, the serum adiponectin level may be an independent protective factor for the cognitive function of patients with SCI (*β* = 0.210, *p* = 0.043).

## 4. Discussions

The main conclusions of our study are as follows: Compared with the control, the serum adiponectin level of SCI patients decreased significantly; higher quartile levels of serum adiponectin have higher MoCA scores, and this correlation is statistically significant. Further regression analysis suggested that the serum adiponectin level can independently predict the cognitive function of patients with SCI, and it may be a protective neurological factor. The advantage of our study is that we reported for the first time the correlation between serum adiponectin levels and short-term cognitive function (3 months) in patients with SCI.

The role of inflammation in SCI has been deeply studied. In the SCI animal model, the native microglia, as well as the macrophages, neutrophils, and lymphocytes recruited from the surroundings, are rapidly activated within a few minutes to a few days after SCI [16]. These activated cells release proinflammatory cytokines and chemokines, amplify the inflammatory response, and inhibit axon regeneration, thereby inducing secondary neuronal damage [17]. In the autopsy of humans, similar results were found in animal models, namely, the aggregation of inflammatory cells after SCI [18]. In a study on SCI biomarkers, researchers found that the level of inflammatory mediators in the blood is 10% of the concentration in CSF, and both can predict the prognosis of SCI [19]. In addition to acute SCI, persistent inflammation can also be found in chronic SCI. Functional genomics has studied the genome-wide changes of inflammation and other immune-related mediators in SCI and found that the significant reduction of natural killer (NK) cell genes and the upregulation of inflammatory genes indicate that inflammation is involved in the course of chronic SCI [20, 21].

Recently, some studies have found that adiponectin may play a role in a series of inflammatory diseases. The study by Shore and his colleagues found in animal models that exogenous adiponectin can reduce allergen-induced airway hyperresponsiveness and attenuate inflammation [22]. In another study, an asthma model was constructed in adiponectin-deficient mice, and it was found that adiponectin-deficient mice had more severe pulmonary artery muscularization and pulmonary hypertension than normal mice [23]. This result further suggested that adiponectin plays a key role in chronic airway inflammation. Research by Frühbeck et al. found that the ratio of adiponectin/leptin in metabolic syndrome is low, leading to an increase in oxidative stress and inflammation. This ratio can be used as a biological marker of metabolic syndrome [24]. Chinese scholars have discovered that adiponectin plays a protective role in coronary heart disease by inhibiting macrophages and reducing inflammation [25]. A study by the University of Tokyo found that adiponectin-deficient psoriasis mice showed increased infiltration of dermal IL-17-derived V*γ*4+*γδ*-T cells, suggesting that adiponectin is involved in the regulation of skin inflammation [26]. Interestingly, recent studies have shown that recombinant adiponectin peptides can improve neurological damage caused by cerebral hemorrhage; the mechanism of which is through the inhibition of drp1/astrocyte-mediated inflammatory activation [27]. The role of adiponectin in more and more inflammatory diseases has been discovered, suggesting that treatment targeting adiponectin may be a new direction for immunotherapy.

However, the role of adiponectin in SCI has not been reported yet. Adiponectin receptor agonist AdipoRon can reduce astrocyte proliferation after SCI, thereby improving the prognosis, suggesting that activating adiponectin signaling is a potential treatment for SCI [28]. O'Brien and his colleagues found that adiponectin is closely related to the metabolic profile of SCI patients, and the mechanism may be that adiponectin can promote mitochondrial biogenesis [29]. Clinical studies have shown that circulating adiponectin may indeed be a potential biomarker for predicting the risk of osteoporosis and fracture in patients with SCI [30].

In addition, adiponectin is also believed to be involved in the maintenance of cognitive function. A study from Brazil suggests that patients with mild cognitive impairment and Alzheimer's disease have significantly lower serum adiponectin levels [31]. A Polish study showed that patients with diabetes and mild cognitive impairment have low serum adiponectin levels, suggesting that adiponectin may be involved in cognitive impairment [32]. Scholars in Pittsburgh found that adiponectin inhibits the activation of microglia in a PPAR *γ*-dependent manner to improve the cognitive function of vascular dementia [33]. However, the role of adiponectin in the cognitive maintenance of SCI patients has not been reported yet.

Our research has the following limitations: (1) no dynamic study of serum adiponectin levels in patients with SCI, (2) no relevant mechanism studies in animal and cell experiments, (3) no verification of our conclusions in more populations, and (4) the baseline data of SCI patients lack assessment of the severity of the disease, which may be an important factor affecting the prognosis. However, we are the first study to report the correlation between SCI cognitive impairment and adiponectin.

## 5. Conclusions

The level of serum adiponectin in patients with SCI is significantly reduced, and it is related to the decline of cognitive function. Serum adiponectin level is an independent predictor of cognitive decline in SCI patients. Our research is helpful for the development of targeted therapy for adiponectin in the future, thereby reducing the socioeconomic burden of the complications of cognitive impairment after SCI.

## Figures and Tables

**Table 1:** Baseline data and serum adiponectin levels of study population.

	Controls (*n* = 64)	SCI (*n* = 92)	*p* value
Age (years)	57.1 ± 6.3	57.6 ± 6.7	0.639
Gender (male/female)	50/14	76/16	0.485
Smoking, *n* (%)	34	51	0.776
Drinking, *n* (%)	42	59	0.848
Diabetes, *n* (%)	11	19	0.589
Hypertension, *n* (%)	15	26	0.679
LDL (mmol/l)	2.4 ± 0.8	2.5 ± 0.9	0.476
HDL (mmol/l)	1.2 ± 0.2	1.1 ± 0.3	0.021
FBG (mmol/l)	5.6 ± 0.7	5.8 ± 0.8	0.108
Adiponectin, (*μ*g/ml)	6.7 ± 0.9	6.1 ± 1.1	<0.001
MoCA (points)	27.4 ± 1.3	23.3 ± 2.5	<0.001

LDL: low-density lipoprotein; HDL: high-density lipoprotein; FBG: fasting blood glucose; MoCA: Montreal Cognitive Assessment.

**Table 2:** Relationship between cognitive impairment and adiponectin levels.

Variable	*Q*1	*Q*2	*Q*3	*Q*4	*p* values
MoCA score	19.1 ± 1.8	21.3 ± 1.7	23.4 ± 2.1	24.8 ± 1.5	<0.001

MoCA: Montreal Cognitive Assessment.

**Table 3:** Analysis of multiple linear risk factors of cognitive impairment in SCI patients.

	Regression coefficient	95% confidence interval	*p* value
Age	0.236	0.204-1.027	0.164
Gender	0.195	0.168-1.053	0.256
Smoking	0.148	0.129-1.076	0.223
Drinking	0.179	0.145-1.098	0.152
Diabetes	0.204	0.187-1.105	0.105
Hypertension	0.183	0.162-1.081	0.203
LDL	0.137	0.110-1.049	0.261
HDL	0.162	0.131-1.024	0.097
FBG	0.221	0.186-1.132	0.238
Adiponectin	0.210	0.153-0.867	0.043

SCI: spinal cord injury; LDL: low-density lipoprotein; HDL: high-density lipoprotein; FBG: fasting blood glucose.

## Data Availability

The study data presented may be made available from the corresponding authors upon reasonable request.
